# A Smart Checkpointing Scheme for Improving the Reliability of Clustering Routing Protocols

**DOI:** 10.3390/s101008938

**Published:** 2010-09-29

**Authors:** Hong Min, Jinman Jung, Bongjae Kim, Yookun Cho, Junyoung Heo, Sangho Yi, Jiman Hong

**Affiliations:** 1 School of Computer Science and Engineering, Seoul National University, Seoul, Korea; E-Mails: hmin@os.snu.ac.kr (H.M.); jmjung@os.snu.ac.kr (J.J.); bjkim@os.snu.ac.kr (B.K.); ykcho@os.snu.ac.kr (Y.C.); 2 Department of Computer Engineering, Hansung University, Seoul, Korea; E-Mail: jyheo@hansung.ac.kr (J.H.); 3 The National Institute for Research in Computer Science and Automatic Control (INRIA) / Montbonnot Saint Martin, France; E-Mail: sangho.yi@inrialpes.fr (S.Y.); 4 School of Computing, Soongsil University, Seoul, Korea

**Keywords:** checkpointing, wireless sensor networks, clustering routing protocols

## Abstract

In wireless sensor networks, system architectures and applications are designed to consider both resource constraints and scalability, because such networks are composed of numerous sensor nodes with various sensors and actuators, small memories, low-power microprocessors, radio modules, and batteries. Clustering routing protocols based on data aggregation schemes aimed at minimizing packet numbers have been proposed to meet these requirements. In clustering routing protocols, the cluster head plays an important role. The cluster head collects data from its member nodes and aggregates the collected data. To improve reliability and reduce recovery latency, we propose a checkpointing scheme for the cluster head. In the proposed scheme, backup nodes monitor and checkpoint the current state of the cluster head periodically. We also derive the checkpointing interval that maximizes reliability while using the same amount of energy consumed by clustering routing protocols that operate without checkpointing. Experimental comparisons with existing non-checkpointing schemes show that our scheme reduces both energy consumption and recovery latency.

## 1. Introduction

Wireless Sensor Networks (WSNs) have recently been considered as an attractive research field and an important computing platform when serving as an infrastructure for implementing pervasive or cyber physical systems [1]. Sensor networks typically are composed of numerous (hundreds or even thousands) sensor nodes that are deployed in the target field and they autonomously construct the desired network. An example of a wireless sensor network application is collecting information from the network’s environment and sending the collected information to a Base Satiation (BS) over the network. To maximize the cost-efficiency of the overall sensor network, each sensor node has limited resources in terms of CPU power, size of memory, and storage capacity. Moreover, this type of network encounters power constraints because sensor nodes need a battery to operate properly [2]. Most previous studies have focused on resource constraints related to real-time features, scalability and energy efficiency of such networks [3].

In WSNs, the communication cost (*i.e.*, the power consumption of the radio module for data transmission among sensor nodes) is much higher than the operation cost (*i.e.*, CPU power consumption). Therefore, routing protocols and data aggregation schemes have been researched to reduce the energy consumed when sending the collected information to the BS. Especially, algorithms that are based on clustering routing protocols are designed to reduce the number of messages sent to the BS from each sensor node by using a hierarchical structure. In this type of scheme, the whole network is divided into several clusters and the network elects one node in each cluster to be called a cluster head. Each cluster head gathers information from its member nodes and performs data aggregation; thus, clustering routing protocols can minimize the number of packets sent to the BS. Through this mechanism, energy efficiency is improved and wireless communication interference problems are mitigated [4]. However, recovery cost and recovery latency increase following communication failure of a cluster head that contains information about all the sensor nodes within the cluster. Such failure occurs frequently because wireless communication sensor nodes have resource constraints and may be deployed in harsh environments.

In this paper, we propose checkpointing of the cluster head as a method of improving reliability and reducing recovery latency of the clustering routing protocols. A cluster head sends routing and collected data information to backup nodes, which periodically save the state of its cluster head. If a cluster head is in transient fault, then one of the backup nodes detects the cluster head failure and a backup node takes on the role of its cluster head. Using checkpointing, the cluster can quickly recover from a transient fault of cluster head by omitting re-election of the cluster head and by preventing loss of the collected information. We also derive the optimal checkpointing interval by considering the failure rate of each node and satisfying the expected reliability requirement. This is the first report of solving this checkpointing interval problem in WSNs and is one of contributions of our paper. If we apply the optimal checkpointing interval to our scheme, reliability is maximized while keeping the same level of energy consumption of clustering routing protocols operating without checkpointing. We evaluate our scheme using network protocol simulation software and implement it to sensor nodes that are run on TinyOS [5].

The paper is organized as follows. In Section 2, we describe previous works related to fault tolerant schemes of wireless sensor networks. Section 3 explains the design of our checkpointing scheme, and Section 4 shows its implementation. In Section 5, we evaluate the impact and performance of our scheme on a resource-constrained sensor network in terms of both energy consumption and recovery latency. A conclusion is presented in Section 6.

## 2. Related Works

This section briefly introduces prior studies related to fault tolerant schemes. We describe the features of each scheme and explain their pros and cons.

### 2.1. Checkpointing the Sink Node

In [6], the authors proposed the concept of in-network fault tolerance for achieving enhanced network dependability and performance. In that scheme, the sink node periodically checkpoints its state and saves it in the memory of one or more sensor nodes, so called checkpoint sensors. When a sink node (*S_1_*) fails or reaches an energy level below its threshold, another sensor node will be selected to operate as the new sink node (*S_2_*). After applying this approach m times, the sink will be located in a sensor denoted by *S_m_*. If the sink is located on *S_m_*, then *S_m−1_* is the checkpoint sensor and the path between *S_1_* and *S_m_* is the checkpoint path. When a sink node (*S_m_*) fails, *S_m−1_* detects the failure and becomes the sink instead; it iteratively operates in this sequence through the checkpoint path. This scheme is simple to implement, but energy consumption and reliability vary according to the position of the sink node.

### 2.2. Checkpointing all Nodes

Each sensor node within a WSN tends to fail because of software (S/W) or hardware (H/W) related failures. To solve this type of problem, different mechanisms have been designed for each sensor node. Some researchers have suggested a checkpointing scheme based on the density of the neighbors [7]. In such a scheme, each node broadcasts the checkpoint packet to its neighbor nodes, and the neighbor nodes decide whether or not to save the checkpoint packet as the density of sensor nodes.

In [8], authors proposed a flash file system that supports the flexible use of storage capacity for a variety of applications. When considering the memory and energy constraints of the sensor nodes, they use an efficient compaction and storage organization techniques. To tolerate software faults in sensor applications, Capsule, an efficient log-structured file system for flash memory provides the necessary checkpointing and rollback of object states. These schemes improve the reliability of the network, but the scalability issue must be considered when these schemes are used.

### 2.3. Macroprogramming

Macroprogramming means that a programmer describes a sensor network application as a centralized program and a compiler then generates the node level program. Gummadi *et al.* designed a simple checkpoint application programming interface (API) for macroprograms and implemented Kairos, a framework that consists of a program language based on Python, a code generator, and a compiler [9]. If we macroprogramming is applied to a sensor application, then the synchronization problem is automatically solved via the Kairos runtime system. Although macroprogramming has many pros, it is inflexible and too complex for some sensor applications, such as those related to forest fire detection and enemy tracing.

## 3. Checkpointing Scheme for Clustering Routing Protocols

In this section, we present the design of a checkpointing scheme for clustering routing protocols in detail. First, the essential concept of the clustering routing protocols and its features is described. Then, the design of our scheme and the model for finding the optimal checkpointing interval are presented.

### 3.1. Clustering Routing Protocol

The main aim of clustering routing protocols (hierarchical protocols) is to efficiently maintain the energy consumption of sensor nodes by involving them in multi-hop communication within a particular cluster and by performing data aggregation in order to decrease the number of messages transmitted to the BS [4]. Since the Low-Energy Adaptive Clustering Hierarchy (LEACH) [10] protocol was proposed, there have been many studies on clustering routing protocols such as PEGASIS [11], TEEN [12], ATEEN [13] and OEDSR [14]. These protocols form clusters of sensor nodes based on received signal strength, and they use cluster heads as routers to send the collected information to the BS.

**Figure 1.**
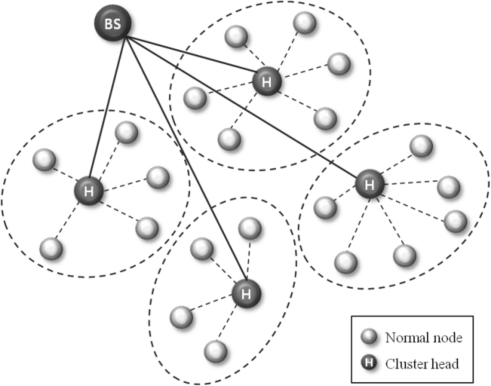
The concept of the clustering routing protocol.

Figure 1 shows the concept of the clustering routing protocol. The depicted network is divided into four clusters, and it elects cluster heads based on the residual energy within each cluster. Normal nodes only communicate with their cluster head, which in turn, aggregates the collected information and sends it to the BS. In this scheme, cluster head failures are more critical than those of normal nodes. When a cluster head fails, re-election of the cluster head is performed within the cluster. Such a recovery scheme is a time and energy consuming process. Therefore, to improve the quality and reliability of sensor networks, a fault tolerant mechanism is needed for such cluster heads.

### 3.2. System Design

We propose a checkpointing scheme for the cluster head in clustering routing protocols that will minimize recovery cost and recovery latency. During the cluster head election step, our scheme elects additional backup nodes for checkpointing the cluster head information. All collected information sent by normal nodes to the cluster head is also saved in the backup nodes. The backup nodes periodically detect the state of the cluster head, and if the cluster head has a transient problem, then one of backup nodes replaces the failed cluster head to play the role of a new cluster head.

**Figure 2.**
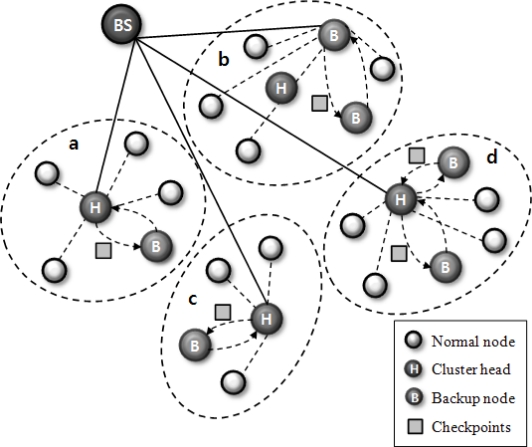
Overview of our scheme.

Figure 2 presents an overview of our scheme applying the cluster head checkpointing mechanism. When the cluster head operates properly (see clusters a, c, d in Figure 2), backup nodes save only the checkpoint information and they monitor the state of the cluster head. In the case of cluster b, the cluster head cannot carry out its tasks when it encounters an S/W or H/W problem. A backup node then operates as a cluster head based on the obtained checkpointing information. Through this checkpointing scheme, we can prevent information loss caused by failure in the cluster head, and we can reduce recovery latency related to the frequent re-election of a cluster head.

In clustering routing protocols, the communication range of a cluster head is larger than that of its cluster. To prevent network partition and orphan node problems, cluster heads adjust their communication ranges properly. In our mechanism, backup nodes can also adjust their communication range to cover all member nodes of their cluster.

### 3.3. System Modeling

We use the Markov model to find the minimum number of backup nodes that meets the expected reliability of users and the energy analysis model to determine the optimal checkpointing interval. Table 1 shows the notations and functions used when modeling our system.

#### 3.3.1. Assumptions

In order to simplify our model, we make the following assumptions:

- the reference network model is based on [15].
- all nodes know their residual energy.
- there are no communication errors between two nodes, and
- failure rate (*λ*) is based on the Poisson distribution.

#### 3.3.2. The minimum number of backup nodes

In our scheme, there is a trade-off between reliability and energy consumption. As the number of backup nodes increases, reliability also increases. However, the energy consumption of the checkpointing process also increases, and, as a result, the life-time of the network decreases. Therefore, we need to find the minimum number of backup nodes that satisfies user reliability expectations (*R_user_*). Here, we apply the Markov model to determine the minimum number of backup nodes when the expected reliability is specified by a user or an application designer.

**Figure 3.**
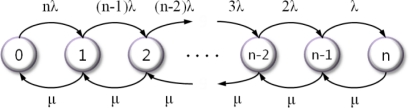
The state diagram of our scheme.

In [16], there is a special case of a birth-death process that reflects that of a continuous-time Markov model. Figure 3 shows the state diagram of our model, where the state indicates the number of failure nodes.

If the failure rate of each node (including the cluster head) is λ and the repair rate is μ, the expressions for steady-state probabilities are obtained via Equations (1) and (2):

(1) $$\pi_{k}=\pi_{0}\prod_{i=0}^{k-1}\frac{\lambda \left(n-i\right)}{\mu },0\leq k\leq n$$

(2) $$\pi_{0}=\frac{1}{\sum_{k=0}^{n}\rho^{k}\frac{n!}{\left(n-k\right)!}}$$

Each node has its own repair facility such as a watchdog timer that monitors the state of the sensor node periodically. If a sensor node has problems and cannot operate properly, a watchdog timer restarts the system. When the watchdog timer interval is the repair rate (*μ*), the availability of an individual component (*A_indiv_*) is obtained via Equation (3), and the steady-state availability (*A_steady_*) is computed via Equation (4):

(3) $$A_{\text{indiv}}=\frac{1}{1+\frac{\lambda }{\mu }}=\frac{1}{1+\rho }$$

(4) $$A_{\text{steady}}=1-\pi_{n}=1-\frac{\rho^{n}n!}{\sum_{k=0}^{n}\rho^{k}\frac{n!}{\left(n-k\right)!}}$$

When A_steady_ equal to the expected reliability of the user (*R_user_*), *μ* is equal to the frequency of watchdog timer and the failure rate of each node, (*λ*), is given, we can define the minimum number of backup nodes (*n−1*) through Equation (4).

#### 3.3.2. Optimal checkpointing interval

**Figure 4.**
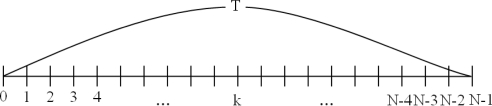
The data collection step.

In the clustering routing protocols, a cluster head is in charge of the data collection activity, and this step is modeled as in Figure 4.

This cluster is composed of N nodes (a cluster head and *N−1* normal nodes), and each member node sends sensing data to its cluster head during time *T*. If the failure rate of each node is *λ*, then e^−λT^ represents a lack of failure for each node during the total time of data collection of all member nodes (*i.e.*, time *T*). In this condition, the probability of failure is P_k_ = (e^−λT^)^k−1^(1 − e^−λT^), when the cluster head gathers data from the *k^th^* node.

To compare the energy consumption of our checkpointing scheme with that of an existing non-checkpointing scheme, we define *E_pre_* and *E_ckpt_* as in Equation (5):

(5) $$\begin{array}{c} E_{\text{pre}}=\sum_{k=0}^{N-1}\left\{\left(1-P_{k}\right)\cdot {\text{MSG}}_{s}\cdot E_{\text{rf}}+P_{k}\cdot \left(E_{\text{elec}}+{\text{MSG}}_{s}\cdot E_{\text{rf}}\right)\right\}\\ E_{\text{elec}}=\left\{{\left(N-1\right)}^{2}+2\left(N-1\right)\right\}\cdot {\text{MSG}}_{s}\cdot E_{\text{rf}}\\ E_{\text{ckpt}}=\sum_{k=0}^{N-1}\left\{\left(1-P_{k}\right)\cdot {\text{MSG}}_{s}\cdot E_{\text{rf}}+P_{k}\cdot {\text{MSG}}_{s}\cdot E_{\text{rf}})\right\}+\left(n-1\right)\cdot {\text{MSG}}_{s}\cdot E_{\text{rf}}\cdot \left\lceil \frac{k}{I_{\text{ckpt}}}\right\rceil \end{array}$$

The energy consumption of the existing clustering routing protocols (*E_pre_*) is divided by two parts. One is the summation of energy consumption of each member node while the cluster head operates properly. The other is the energy consumption of the recovery process. In clustering routing protocols without a checkpointing mechanism, when a cluster head fails, member nodes re-elect a new cluster head. This recovery process includes many types of messages such as a recovery process start message (*N − 1*), broadcasting the remaining energy notification messages of normal nodes (*(N − 1)^2^*), and a recovery process end message of the new cluster head (*N − 1*), used for finding member nodes and constructing a routing table [17]. The energy consumption of the cluster head re-election process is represented by *E_elec_*.

The energy consumption of a clustering routing protocol with checkpointing (*E_ckpt_*) is similar to that of previously reported clustering routing protocols. However, the proposed checkpointing scheme excludes re-election cost (*E_elec_*) because our scheme does not need to re-elect a new cluster head, although it does includes checkpointing costs during time *k*.

Algorithm 1 explains the checkpointing and recovery process of our scheme. As our scheme can omit cluster head election and state recovery, it reduces energy consumption and recovery latency.

**Algorithm 1.** the recovery process of our scheme.

**if** cluster head failure is detected != true **then**
**if** elapsed time >= *I_ckpt_***then**
checkpointing in backup nodes
**else**
collecting data from normal nodes
**end if**
**else**
one of the backup nodes is assigned as
a new cluster head
broadcast ID of a backup node to its normal nodes
**end if**

The optimal checkpointing interval is the time between two successive checkpoints while satisfying the *E_pre_* ≥ *E_ckpt_* condition. This condition means that the checkpointing energy is to be less than the re-election energy. Therefore, the minimum value of *I_ckpt_* is the optimal checkpointing interval, which is derived through Equation (6):

(6) $$\begin{array}{r} E_{\text{pre}}\geq E_{\text{ckpt}}\;\;,\;\;I_{\text{ckpt}}>0\\ E_{\text{elec}}\geq {\text{MSG}}_{s}\cdot E_{\text{rf}}\cdot \left(\frac{\lambda T}{I_{\text{ckpt}}}\right)\\ I_{\text{ckpt}}\geq \frac{\lambda T}{{\left(N-1\right)}^{2}+2\left(N-1\right)} \end{array}$$

As recovery latency is in direct proportion with the number of required messages, we compare the recovery latency of our checkpointing scheme with that of previous schemes through Equation (7). In clustering routing protocols without checkpointing, the recovery latency includes the cluster head re-election process and the scheduling latency of the ZigBee Medial Access Control (MAC) protocol [15]. In our proposed scheme, backup nodes wait one checkpointing interval (*I_ckpt_*) for detection of a cluster head failure, and a backup node sends its identification (ID) code to member nodes to commit that node to the role of its cluster head:

(7) $$\begin{array}{c} D_{\text{pre}}=\left\{{\left(N-1\right)}^{2}+2\left(N-1\right)\right\}\cdot D_{\text{schd}}\\ D_{\text{ckpt}}=I_{\text{ckpt}}+\left(N-1\right)\cdot D_{\text{schd}} \end{array}$$

## 4. Implementation

**Figure 5.**
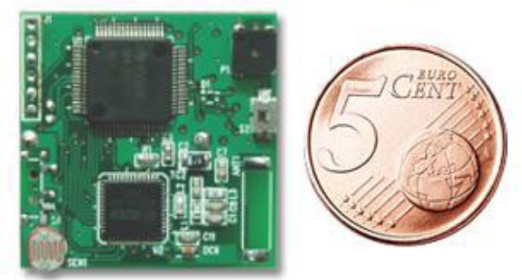
The target sensor node: Ubi-coin.

We have implemented our checkpointing scheme for clustering routing protocols to evaluate recovery latency in a real world situation. Figure 5 shows an example of the target sensor node called Ubi-coin, and Table 2 describes the H/W specifications of the sensor node. We implement our scheme using the TinyOS API, a well-known sensor operating system in wireless sensor networks (available at http://www.tinyos.net/). The testbed is composed of 50 nodes that include a cluster head, three backup nodes, and 46 member nodes. This testbed represents a single cluster of a sensor network in which there are several clusters.

To simplify the testbed, all nodes were able to communicate with each other within a one-hop range and we changed the number of nodes range from 10, 20, and 50. Each node periodically collects temperature data through a temperature sensor and sends the obtained data to the cluster head in the order of its ID code.

## 5. Performance Evaluation

We evaluate our scheme in terms of energy efficiency and recovery latency. Table 3 describes the parameters used for the evaluation. The value of the parameters are based on [15] and [19], studies that researched energy consumption and communication latency in WSNs.

**Figure 6.**
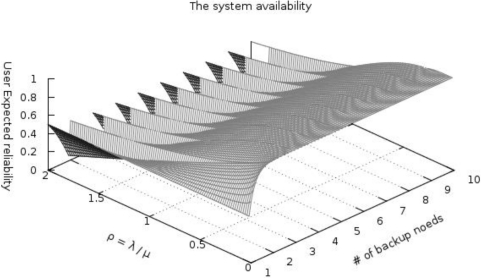
The steady-state availability of our scheme.

To compare energy consumption between clustering routing protocols without checkpointing and with checkpointing, the number of backup nodes needs to be determined. Figure 6 shows the steady-state availability (*A_steady_*) of our scheme, the number of backup nodes, and the ratio ρ (*i.e.*, *λ/μ*) obtained by plotting Equation (4). When the failure rate (*λ*) is higher than the repair rate (*μ*) of the watchdog timer (ρ > 1), ant system availability is dramatically decreased because the value of Equation (4) exponentially increases and decreases by ρ. To improve availability, the watchdog timer interval must be appropriately decreased. If watchdog timer rate is higher than the failure rate, resulting in ρ < 1, the reliability of the system is more than 80% when using three backup nodes. In case of the repair rate is the same to the failure rate (ρ = 1), and our system provides reasonable availability (more than 73%) when using just three backup nodes. We have assumed ρ is smaller than 1 in order to satisfy user expected reliability (*R_user_*) requirements. Under those conditions, three backup nodes are sufficient to satisfy the system availability requirements.

**Figure 7.**
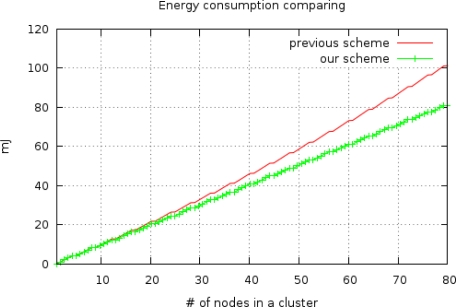
Energy consumption of non-checkpointing (*E_pre_*) and checkpointing (*E_ckpt_*).

**Figure 8.**
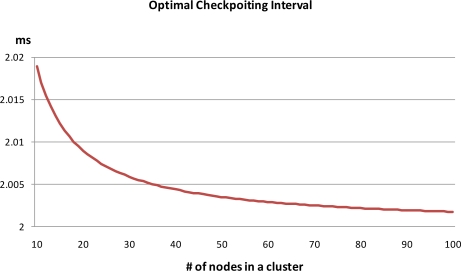
Optimal checkpointing interval.

The energy consumption between clustering routing protocols without checkpointing (*E_pre_*) and with checkpointing (*E_ckpt_*) is compared via Equation (5) with the results shown in Figure 7. In this comparison, three backup nodes request the checkpoint packet from the cluster head whenever member nodes send sensing data to the cluster head, with *I_ckpt_* = 17 ms. The energy consumption of the non-checkpointing scheme is higher than that of our scheme and the difference of two schemes steadily increases with increases in the number of nodes in a cluster. By using this extra energy, our scheme can reduce the check pointing interval and increase the reliability of sensor network. In this case, we derived optimal checkpointing intervals of between 2.019 ms and 2.002 ms, when the number of sensor nodes ranged from 10 to 100 (Figure 8). The results show that as the number of sensor nodes increase, the amount of extra energy (*E_pre_* − *E_ckpt_*) is increase, and the amount of checkpointing messages also increase. In summary, the optimal checkpointing interval approaches 2ms as the number of sensor nodes in a cluster increases.

**Figure 10.**
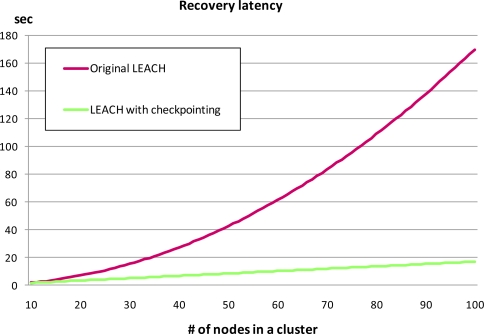
Recovery latency comparison between checkpointing and non-checkpointing LEACH by obtained using the GloMoSim simulator.

**Figure 11.**
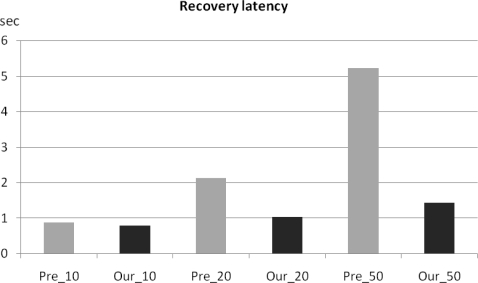
Recovery latency comparison between checkpointing and non-checkpointing LEACH results by using a real-world testbed.

We tested our checkpointing scheme on the aforementioned testbed to evaluate recovery latency. Figures 10 and 11 show the recovery latency comparison between our checkpointing scheme applied to LEACH and that from the original LEACH with the results obtained via GloMoSim and a real-world testbed respectively. Simulation result shows the recovery latency of the original LEACH increases exponentially while that from LEACH with our checkpointing scheme applied increased more slowly and steadily (Figure 10).

Recovery latency is affected by the amount of messages sent during the recovery process. In the original LEACH, *O(n^2^)* messages are generated during the re-election process as the number of nodes increases in a cluster. However, LEACH with our checkpointing scheme applied generates only *O(n)* messages via the a backup node; thus, recovery latency with checkpointing increases linearly.

During implementation testing, we uniformly deployed sensor nodes in a 10 m × 10 m test field and created failure conditions by turning off the cluster head, or blocking wireless communication by using obstacles. We then measured the completion time for data collection from all member nodes within a cluster and calculated the mean recovery latency time after running the conditions 10 times. The implementation results (Figure 11) were similar trend to simulation result in Figure 10. As in the simulation results, the implementation results showed that recovery latency using our checkpointing scheme steadily increases, while that of the original LEACH increases exponentially. Therefore, our scheme is also more efficient than previous clustering routing protocols without checkpointing in terms of energy consumption and recovery latency.

## 6. Conclusions

When designing an efficient sensor application, we must consider the resource constraints of sensor nodes and their scalability. WSN users are concerned about information quality and user requirements for real-time features are also increasing. Moreover, sensor applications are expanding into harsher and more dangerous environments. Therefore, fault tolerant schemes have emerged as important issues in WSNs.

Clustering routing protocols such as LEACH, PEGASIS and TEEN were designed to improve both energy efficiency and scalability. These protocols compose clusters and elect a cluster head in each cluster. The cluster heads aggregate data from its member nodes and reduce the amount of messages sent by member nodes to the BS directly. In clustering routing protocols, cluster head management is needed because the role of the cluster head is more important than one of member nodes.

In this paper, we proposed a checkpointing scheme for clustering routing protocols. Our scheme can reduce energy consumption and recovery latency when a cluster head fails transiently. In addition, our checkpointing scheme is easy to implement. The simulation and real-world testbed results show energy consumption and recovery latency efficiencies when our checkpointing scheme is implemented.

## Figures and Tables

**Figure 9.**
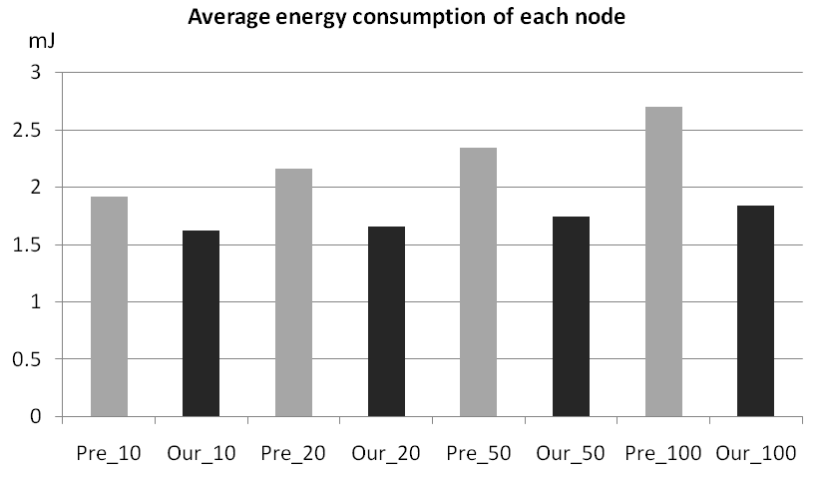
Comparing average energy consumption of selected node group sizes.

**Table 1.** List of notations.

**Notation**	**Description**

*N*	The number of nodes in a cluster
*n*	The number of backup nodes +1 (a cluster head)
*λ*	Failure rate of each node
*μ*	Repair rate of backup nodes
*ρ*	*λ/μ*
*π_k_*	Steady-state probability of state *k*
*R_user_*	User Expected reliability
*MSG_s_*	Message length
*E_elec_*	Energy consumption by cluster head election
*E_init_*	Initial residual energy of a node
*E_rf_*	Communication cost between two nodes
*I_ckpt_*	Checkpointing interval
*T*	Total time of collecting data from all member nodes
*t*	Elapsed time of collecting data form a member node ( *t = T / (N−1)* )
*E_pre_*	Energy consumption of clustering protocols without checkpointing
*E_ckpt_*	Energy consumption of clustering protocols with checkpointing
*D_schd_*	Packet scheduling delay
*D_pre_*	Recovery latency of previous scheme
*D_ckpt_*	Recovery latency of our scheme

**Table 2.** Hardware specifications.

**Component**	**Description**

Microprocessor	MSP430 F1611
RAM	10Kbye
Flash	48Kbyte + 256Byte
LED	Full color LED 1ea
Power	3V DC
RF	CC2420

**Table 3.** Parameters for simulation.

**Parameters**	**Value**

Field size	500 m × 500 m
N	10, 20, 50, 100
n	3
λ	10^−4^ (0 < λ < 1.0)
μ	2*10^−4^, 2*10^−6^ (0 < μ < 1.0)
ρ	0.5 (λ/μ)
R_user_	0.8 (80%)
MSG_s_	128 Bytes
E_init_	0.5 J
E_rf_	80 nJ
I_ckpt_	17 ms ≥ I_ckpt_ ≥ 0 ms
D_schd_	17 ms
T	(N−1) * D_schd_
